# Pomegranate Peel as Suitable Source of High-Added Value Bioactives: Tailored Functionalized Meat Products

**DOI:** 10.3390/molecules25122859

**Published:** 2020-06-21

**Authors:** Patricia Gullón, Gonzalo Astray, Beatriz Gullón, Igor Tomasevic, José M. Lorenzo

**Affiliations:** 1 Nutrition and Bromatology Group, Department of Analytical and Food Chemistry, Faculty of Food Science and Technology, University of Vigo, Ourense Campus, 32004 Ourense, Spain; pgullon@uvigo.es; 2 Department of Physical Chemistry, Faculty of Science, University of Vigo (Campus Ourense), As Lagoas, 32004 Ourense, Spain; gastray@uvigo.es; 3 CITACA, Agri-Food Research and Transfer Cluster, Campus Auga, University of Vigo, 32004 Ourense, Spain; 4 Department of Chemical Engineering, Faculty of Science, University of Vigo (Campus Ourense), As Lagoas, 32004 Ourense, Spain; bgullon@uvigo.es; 5 Department of Animal Source Food Technology, University of Belgrade, Faculty of Agriculture, Nemanjina 6, 11080 Belgrade, Serbia; tbigor@agrif.bg.ac.rs; 6 Centro Tecnológico de la Carne de Galicia, Rúa Galicia No 4, Parque Tecnológico de Galicia, San Cibrao das Viñas, 32900 Ourense, Spain; 7 Área de Tecnología de los Alimentos, Facultad de Ciencias de Ourense, Universidad de Vigo, 32004 Ourense, Spain

**Keywords:** pomegranate, bioactive compounds, meat products, oxidative stability

## Abstract

In the last few years, the consumer’s concern with the relationship between health and diet has led to the search of foods with functional properties beyond the nutritional. In this framework, the consumption of pomegranate has increased due to their sensorial attributes and remarkable amounts of bioactive compounds, which generate, at the same time, huge amounts of by-products. A search in the Scopus database for the last 10 years has revealed the rising interest in pomegranate peel (PP), the main residue from this fruit. The meat industry is a food sector that has had to search for new alternatives to substitute the use of synthetic preservatives by new natural additives, to extend the self-life and keep the quality attributes of their processed products. This review sets out the main bioactivities of PP extracts, and their incorporation in meat products is elaborated. PP is a good source of bioactive compounds, including phenolic acids, flavonoids and hydrolyzable tannins, which have beneficial health effects. It can be concluded that the reformulation of meat products with PP extracts is a suitable strategy for enhancing their technological characteristics, in addition to conferring functional properties that make them healthier and potentially more acceptable for the consumer.

## 1. Introduction

In western diets, meat and meat products are one of the main sources of high-biological value protein, in addition to containing micronutrients such as minerals (iron, magnesium, potassium, selenium and sodium) and vitamins (A, B_12_, folic acid, among others) that are highly bio-available [1]. Despite these excellent nutritional properties, the intake of meat and meat products is related with a higher incidence of cardiovascular diseases and obesity, increasing the negative perception attached to the consumers of these food products in recent years [2]. On the other hand, due to its rich nutritional profile, the meat presents a high susceptibility to deterioration due to the microbial growth and oxidation processes that take place when the muscle is transformed in the meat during the meat processing and storage [3,4,5,6]. To overcome these drawbacks, and in line with the rising awareness by consumers of the relationship between diet and health [7], in the last few years, the meat industry has had to face the double challenge of offering more healthy meat processed products, and guaranteeing their stability [8,9,10,11].

Until now, synthetic antioxidants, such as butylated hydroxytoluene (BHT) and butylated hydroxyanisole (BHA), have been widely used in the food industry as preservative agents to extend the self-life of food products, but their incorporation should be reduced due to their harmful effects on health, as described in in vitro and in vivo studies [4]. The need to find alternatives to substitute the synthetic additives has led to scientific researchers and meat industries devoting great efforts to using renewable biomass as a natural source of new biomolecules with functional properties [7,12,13,14,15]. In this context, the agri-food industry produces large amounts of waste and by-products that usually are underutilized or discarded, but that contain remarkable amounts of bioactive compounds with properties that make them suitable to be used as natural ingredients [16,17,18,19]. Therefore, this strategy outlines a promising approach that will allow the removal of a residue without economical value through the recovery of extracts rich in biomolecules, that could then be incorporated in the food chain, contributing to a circular economy model [20,21,22,23].

Due to the therapeutic properties associated with pomegranate, its consumption in the form of fresh fruit, juices, jams or dietary supplements has increased significantly in the last decade [5,7,8,12]. The industrial processing of this fruit generates huge amounts of by-products, mainly peels and seeds, which usually are discarded as waste without any valorization [24]. Among these residues, pomegranate peel (PP, around 40–50% of the total fruit weight) is an excellent source of phenolic compounds (flavonoids, phenolic acids and tannins), protein and bioactive peptides, and polysaccharides, among others [7,24]. Several in vitro bioactivities, such as antioxidant, anti-inflammatory, antitumor and antiproliferative activities, have been described for PP extracts in the literature [25,26,27,28]. The wide variety of biomolecules with different functionalities suggests that PP extracts could be used in the meat industry as a functional ingredient [5,9,11,12]. In this sense, the reformulation of meat products with extracts from PP could be an alternative for solving the mentioned inconveniences, and it opens the possibility of formulating customizable meat products tailored to the requirements of certain population groups. Due to this, a growing interest in PP is reflected in the research studies concerning this residue.

A key point in the obtaining of biomolecules from agri-food residues is the selection of the adequate extraction technology, since it should be efficient and should maintain the bioactivities in order to guarantee their functionality when they are incorporated in a food matrix [2]. Traditionally, conventional methods based on organic solvents and their aqueous mixtures have been widely used in the recovery of phytochemicals from agri-food sources. However, due to the fact that they present disadvantages that negatively affect the bioactive properties of the extracted biomolecules, in the last few years, eco-friendly and sustainable avant-garde technologies have been developed and applied in combination with smart solvents, namely deep eutectic solvents and natural deep eutectic solvents, in the field of the recovery of natural functional ingredients. Among these techniques, microwave-assisted extraction (MAE), ultrasound-assisted extraction (UAE), pressurized liquid extraction (PLE) and supercritical fluid extraction (SFE) are being used in the recovery of biomolecules from PP [23,25,29,30].

This review collects the research advanced in the last 10 years on the potential use of PP as a suitable source of high-added value biocompounds for formulating tailored functionalized meat products. Aspects such as the main phytochemicals present in PP and their bioactivities are revised. The role of the extracts, in both the technological and health properties of reformulated meat products, are also featured.

## 2. Main Bioactive Constituents Present in Pomegranate Peel

**Figure 1:**
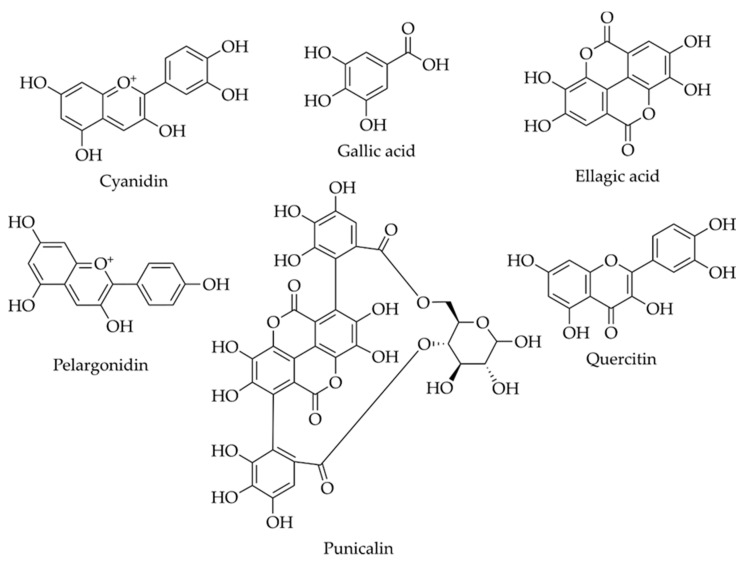
Chemical structure of some biomolecules identified in pomegranate peel [26].

It has been described that PP is an excellent source of valuable biocompounds, including phenolic acids (hydroxycinnamic and hydroxybenzoic acids), flavonoids (anthocyanins, catechins and other complex flavonoids) and hydrolyzable tannins (ellagic and gallic acids, pedunculagin, punicalin and punicalagin), all of them with proven beneficial health effects [26,27,31]. Besides, pomegranate by-products also contain organic acids, minerals (calcium, phosphorus, magnesium, potassium and sodium), protein and fatty acids (mainly punicic, linoleic and oleic acids present in the seeds) [27,31]. Some of the main bioactive components are briefly described next. The chemical structures of the main biomolecules identified in PP are displayed in Figure 1. 

### 2.1. Primary Metabolites

#### 2.1.1. Polysaccharides

Despite the fact that most of the research has focused on the phenolic compounds obtained from PP, in recent years this by-product has also been highlighted as a rich source of bioactive polysaccharides. An extensive literature review has revealed that pectin is the major polysaccharide, accounting approximately 20–25% of PP [32]. Pectin is a mixture of polysaccharides made up of galacturonic acid units linked by a bond α(1→4), with three main structures: homogalacturonan (HG), rhamnogalacturonan I (RG-I) and rhamnogalacturonan II (RG-II) [33]. Studies on PP polysaccharides are, to date, scarce, particularly those about their specific composition among the different varieties of pomegranate [34]. Gavlighi et al. [35] reported that the isolated polysaccharides from PP of an Iranian variety (unspecified) contained uronic acids in the range 19.9–30.8%, and neutral sugars, namely glucose (44.9–68.1%), galactose (14.6–19.4%), mannose (3.4–18.1%), arabinose (3.1–18.1%) and rhamnose (3.5–6.0%). The extracted pectin from PP of a “Gabsi” cultivar from Tunisia presented total neutral sugar and galacturonic acid levels that varied from 16.1% to 32.6%, and from 37.7% to 75.5%, respectively [36]. In another study, Shakhmatov et al. [37] indicated that fragments of highly methyl-esterified and low acetylated 1,4-α-d-galactopyranosyluronan mainly constituted the structure of the polysaccharides of PP.

PP has also been described to be a new source of dietary fiber, specifically β-glucans [38]. β-glucan is a heterogeneous non-starch polysaccharide constituted of β-d-glucose units linked by β-(1–4) and β-(1–3) glycosidic bonds [39]. We have only found one study that refers to β-glucans from PP [38]. In that work, the authors obtained 2.4% of solubilized β-glucans from PP of the Akko variety.

#### 2.1.2. Proteins and Amino Acid

The data on the protein and amino acids in PP are limited, and only a handful of research studies have been found on this topic [29]. The protein content in peel was reported to be approximately 3% [40,41]. However, this fraction has been found in a higher content in pomegranate seeds, reaching about 14% [41]. Regarding amino acid levels, these same authors indicated that PP had a higher content of essential amino acids, in particular, lysine, leucine, phenylalanine, tyrosine, threonine and valine, in comparison with the reference protein pattern of Food and Agriculture Organization of the United Nations/World Health Organization (FAO/WHO).

On the other hand, in the last 10 years, there has been an increasing interest in the search for new natural sources for obtaining proteins and bioactive peptides, due to their important beneficial health effects. In this line, the Hernández-Carroto group has isolated, and identified by High-Performance Liquid Chromatography Coupled to Electrospray Ionisation and Quadrupole Time-of-Flight (HPLC-ESI-Q-TOF), several peptides from the PP protein [29,42].

### 2.2. Secondary Metabolites

#### 2.2.1. Phenolic Acids

PP contains a significant amount of phenolic acids, such as gallic, ellagic, vanillic, caffeic, ferulic, cinnamic and p-coumaric acids [26,43]. Both the concentration and the phenolic profile of pomegranate by-products may vary among different pomegranate cultivars [26,31]. For instance, levels of ellagic acid of 7.3 and 16.5 mg/g were quantified in extracts of peel from Tunisia [44] and Spain [45] cultivars, respectively. A medium total content of ellagic acid of 8.4 mg/g was determined in PP samples of different Italian varieties [46]. In peel extracts of an Egyptian pomegranate (Wonderful variety), the main phenolic acids identified and quantified were ellagic acid (12.56 mg/g), gallic acid (2.5 mg/g), cinnamic acid (2.5 mg/g), chlorogenic acid (1.56 mg/g) and coumarin acid (0.91 mg/g) [43]. In another study conducted by Yan et al. [47], six Chinese pomegranate cultivars were analyzed by Ultra-Performance Liquid Chromatography Coupled with Mass Spectrum (UPLC-DAD/ESI-MS), and they reported that the mean content of gallic acid and ellagic acid in peels was 0.57 mg/g and 1.34 mg/g, respectively. Li et al. [48] found higher levels of these compounds (2.59 mg/g of gallic acid and 2.83 mg/g of ellagic acid) in peel extracts obtained from pomegranates collected from a different area of Shaanxi Lintong (China).

Using HPLC-DAD-ESI/MS, Ambigaipalan et al. [49] revealed the presence of 15 phenolic acids in the PP of a pomegranate variety grown in California. Phenolic acids were found mostly in the insoluble-bound form (around 2743 µg/100), compared to esterified and free forms (around 171 µg/100 and around 7 µg/100, respectively).

#### 2.2.2. Flavonoids

Different flavonoids, including catechin, epicatechin, quercetin, rutin, kaempferol, hesperidine, anthocyanins and procyanidins, have been identified in PP, this by-product being considered an excellent source of these phytochemicals [26]. As in the case of phenolic acids, the relative amounts and compositions of flavonoids depend on the pomegranate cultivar, the part of the fruit, the ripeness stage and the storage of the fruit [26,50]. For example, Masci et al. [51] found a higher total flavonoid content (TFC) in ethyl acetate extracts from the peel [0.881 mmol Rutin Equivalents (RE)/g] compared to the total fruit (0.561 mmol RE/g) in pomegranates of Italian origin. In another study performed by Russo et al. [46], catechin content ranged from 0.89 to 11.7 mg/g in methanolic extracts of peel from six Italian pomegranate varieties, while in their respective pulps this value was much lower, ranging from 0.015 to 0.234 mg/g. In these cultivars, PP presented a mean content of rutin of 4.5 mg/g, and this was not detected in the pulps. Recently, El-Hadary and Ramadan [43] determined that hesperidin (5.047 mg/g) was the majority flavonoid, followed by quercetin (3.519 mg/g), in Egyptian PP extracts. Some authors have reported that prolonged storage affects the stability of the flavonoids in pomegranate fruit. Thus, Mphahlele et al. [50] studied how the packaging systems influence the content of bioactive compounds of the whole pomegranate fruit, observing a 65% loss of rutin in the peel after 3 months of cold storage inside a polyliner bag.

Among the different flavonoids found in pomegranate fruit, the anthocyanin group is considered one of the most important, and, together with hydrolysable tannins, it is one of the most valuable biomolecules found in this fruit [52]. They are the main water-soluble pigments responsible for the orange, red and purple colors of the pomegranate fruit [53]. In particular, the peel fraction contains about 30% of the total pomegranate fruit anthocyanins [54]. Many studies have tried to identify and quantify the different types of anthocyanins in pomegranate varieties from several regions of the world, using advanced analytical techniques [31]. Abid et al. [55], using Liquid Chromatography–Tandem Mass Spectrometry (LC-MS-MS), confirmed the presence of pelargonidin-3-pentoside, cyanidin-3-rutinoside, cyanidin-3-glucoside and cyanidin -3-pentoside in peels from four Tunisian ecotypes. HPLC-DAD-ESI-TOF/MS allowed the identification of eight different anthocyanins, namely: (i) pelargonidin-3-glycoside, (ii) pelargonidin-3,5-diglycoside, (iii) delphinidin-3-glycoside, (iv) delphinidin-3,5-diglycoside, (v) cyanidin-3-glycoside, (vi) cyanidin-3,5-diglycoside, (vii) cyanidin -3-pentoside and (viii) cyanidin-3-rutinoside in a Tunisian PP variety called “Nana” [56]. The peel extract from a Spanish pomegranate cultivar named “Mollar de Elche”, collected from an orchard in Acireale (Italy), showed high levels of cyanidin 3-glucoside (49.36%), pelargonidin 3-glucoside (24.62%) and cyanidin 3,5-diglucoside (12.41%) [54]. Other anthocyanins, such as pelargonidin 3,5-diglucoside, delphinidin 3-glucoside and delphinidin 3,5-diglucoside, were detected in much lower concentrations [54].

#### 2.2.3. Tannins

Pomegranate fruit is also known to be an excellent source of hydrolyzable tannins, mainly ellagitannins and gallotannins [26]. In PP, hydrolyzable tannins are mainly found in the form of punicalagin (punicalagin α and β isomers), accounting for about 85% of total tannins [31]. Other tannins detected in pomegranate fruit peels include punicalin, pedunculagin, granatin A, granatin B, corilagin, tellimagrandin, gallagyl hexoside, etc. [26,31]. Using HPLC-DAD-ESI-MS, Ambigaipalan et al. [49] identified 35 hydrolyzable tannins, of which six (monogalloyldiglucose, 2 punicalagin isomers, trigalloylglucopyranose, tetragalloylglucopyranose, pentagalloylglucopyranose) were found for the first time in extracts of PP grown in California. In another study, Wafa et al. [56] detected by HPLC-ESI-TOF/MS the presence of seven ellagitannins, namely (i) punicalin, (ii,iii) granatin A and B, (iv) lagerstannin, (v) pedunculagin, (vi) HHDP-hexoside and (vii) punigluconin, in the peels of the “Nana” variety of pomegranate from Tunisia. Besides, Abid et al. [55] analyzed the tannin composition of PP from four Tunisian ecotypes, and they identified the presence of a castalagin derivative and galloyl-bis-HHDP-hex derivative (casuarinin) in only one of the four cultivars. The content of the tannins also varies between different pomegranate cultivars. For example, in pomegranate fruits of Egyptian origin, the punicalagin concentration in aqueous methanol extracts of peels was reported to be 98.02 mg/g [43], while pomegranates from Israel presented a considerably higher content, at about 612.8 mg/g [57]. Punicalagin concentration ranged from 181 to 255 mg/g in the peel from six Spanish pomegranate cultivars [45].

Among the different parts of the pomegranate fruit, the peel contained the highest content of hydrolyzable tannins compared to the juice or seeds [58]. These authors also indicated that the content of hydrolyzable tannins in a Spanish pomegranate cultivar “Mollar de Elche” gradually decreased during the ripening stages of the fruit. In particular, they found a drop in these compounds of 48% from the beginning to the end of fruit ripening.

Some of the most commonly used technologies to extract bioactive compounds from pomegranate peel, as well as the analytical methods applied for its determination, are compiled in Table 1.

## 3. Biological Activities of Pomegranate Peel

As mentioned previously, PP contains a diverse group of bioactive molecules, with a wide array of biological actions and recognized therapeutic properties, including antioxidant, antimicrobial, anti-inflammatory, antihyperglycemic, antihyperlipidemic, anticancer, etc. [26,28,42,44].

### 3.1. Antioxidant Activity

Natural antioxidants are being increasingly used in the food industry as substitutes for less safe synthetic antioxidants [7,65]. In this context, the antioxidant activity of PP extracts has been widely reported in the literature. Ismail et al. [66] tested the antioxidant capacity of different extracts (aqueous, 70% ethanol, 70% methanol, 70% acetone) of a Pakistani pomegranate variety named “Sufaid Alipuri”. The highest total phenolic content (TPC) was found in extracts from acetone (427.2 mg GAE/g extract), followed by methanol (367.9 mg GAE/g extract), ethanol (361.8 mg GAE/g extract) and water (273.5 mg GAE/g extract). Acetone extracts also showed the highest antioxidant activity, as determined by FRAP assay (91.40 mmol Fe/g), while methanolic extracts showed the highest antioxidant activity by the DPPH (α,α-Diphenyl-β-picrylhydrazyl) method [32 mg ascorbic acid equivalent per gram (mg AAE/g)]. In another study, El-Hadary and Ramadan [43] reported that a pomegranate methanolic extract exhibited a strong antioxidant activity, measured by the methods of DPPH (93.97%) and ABTS (2,2’-azino-di(3-ethylbenzothiazoline-6-suslfonic acid)) (90.92%), and a total phenolic content (TPC) of approximately 189 mg GAE/g extract. Fazio et al. [38] found an IC50 value of 0.58 μg/mL, by DDPH radical, in acetone extracts from PP, whereas for the TROLOX (6-hydroxy-2,5,7,8-tetramethylchroman-2-carboxylic acid) used as a standard, it was 0.89 μg/mL. On the contrary, these extracts showed a lower presence via ABTS radical, finding values of 8.02 vs. 4.06 μg/mL [38].

### 3.2. Antimicrobial Activity

The resistance of many microorganisms to available antibiotics is a major concern worldwide. This, alongside growing consumer interest in “natural food products”, has encouraged researchers and food industries to search for new alternative compounds that can inhibit a broad spectrum of microorganisms [67,68]. In this line, PP has been reported in several studies to be an important source of antimicrobial agents that can protect foodstuffs against spoilage microorganisms, as well as minimizing the occurrence of foodborne illnesses [26]. Gullón et al. [69] demonstrated that a methanolic extract in the range of 30–60 mg/mL was effective against *Staphylococcus aureus*, *Listeria monocytogenes*, *Listeria innocua*, *Escherichia coli*, *Pseudomonas aeruginosa* and *Salmonella* sp. Other studies revealed that acetone extracts are more active than those obtained with methanol, ethanol and water, with inhibition zones of 21.3, 19.4, 17.5 and 11.6 mm, respectively, against *Bacillus subtilis* [66]. According to Wafa et al. [56], an ethanolic extract of Tunisian PP was active against *Salmonella* Kentucky isolated from chicken meat, giving minimum inhibitory concentration (MIC) and minimum bactericidal concentration (MBC) values of 10.75 and 11.5 mg/mL, respectively. In an interesting investigation conducted by Nur Hanani et al. [70], PP was used to develop an active packaging system to prevent microbial spoilage in food products. The results indicated that this film exhibited an inhibitory effect against all the tested bacteria, namely *Listeria monocytogenes*, *Bacillus cereus*, *Escherichia coli* and *Salmonella typhimurium*. The authors attributed this antimicrobial potential to the high phenolic compounds content in PP, especially tannins. These compounds can exert their antimicrobial activity through the precipitation of membrane proteins, causing microbial cell lysis [68].

### 3.3. Other Beneficial Health Properties

Several studies have highlighted the potential of PP extracts to prevent cardiovascular diseases associated with diabetes, hyperlipidemia and hypertension. Ambigaipalan et al. [49] indicated that PP extracts could have an antidiabetic potential, related to the α-glucosidase inhibitory effect. This same finding was also reported by Arun et al. [71], who found that purified fractions of methanolic extracts of PP improved glucose uptake. In this last work, the authors also demonstrated that this fraction was more effective in inhibiting low-density lipoprotein (LDL) oxidation than the ascorbic acid used as a reference (IC50 for pomegranate extract was 16.2 mg/mL vs 24.3 mg/mL for ascorbic acid). In an in vivo study conducted in both diabetic and hyperlipidemic rats, oral administration of a hydro-methanolic extract from PP, at a dose of 200 mg/kg for 56 days of treatment, significantly reduced glucose levels, glycated hemoglobin, total lipid, total cholesterol, LDL cholesterol and very low-density lipoprotein (VLDL) cholesterol, and raised the high-density lipoprotein (HDL) cholesterol levels [43]. Furthermore, the authors also noted that the administration of this natural extract improved liver and kidney functions in comparison with drugs commonly used to treat these diseases.

PP also contains phytochemicals with the capacity to inhibit the activity of the angiotensin converting enzyme (ACE) that is implicated in blood pressure regulation in the renin–angiotensin system [26]. Arun et al. [71] confirmed that an extract rich in polyphenols (mainly gallic acid, p-coumaric acid, cinnamic acid, caffeic acid and chlorogenic acid) was effective as an ACE inhibitor. More recently, Hernández-Corroto et al. [29,42] attributed this antihypertensive capacity of PP to the presence of proteins and peptides.

Some authors have confirmed that PP extracts play an important function in the inactivation of tyrosinase activity involved in the melanogenesis process (melanocyte proliferation and melanin synthesis) [34,72,73]. In a recent study, Laosirisathian et al. [73] reported that an ethanolic extract from PP had higher tyrosinase inhibitory activity than the kojic acid used commercially as a whitening agent (IC50 for the extract was 0.10 µg/mL, and for kojic acid was 7.88 µg/mL).

Cancer has become a leading cause of death in the 21^st^ century. Considering the important impact of this disease, both in terms of human lives and in costs for health systems, there is a growing interest in the development of new potential therapies that can prevent, delay or inhibit cancer progression. Recently, some researchers have also reported the ability of PP extracts to inhibit the growth of tumor cells [74]. Among the active agents present in the PP, the anticarcinogenic effects are mainly attributed to punicalagin, ellagic acid and gallic acid [26]. Li et al. [44] tested, in a BCPAP tumor-bearing mice model, the antitumor efficacy of a PP extract rich in punicalagin and ellagic acid against thyroid cancer. The authors found that PP extract administration at a dose of 125 mg/kg/day led to a 69.8% decrease in tumor growth. Deng et al. [75] also demonstrated that punicalagin and ellagic acid induced apoptosis in two human prostate cancer cell lines (DU145, PC3) and in a mouse prostate cancer cell line (TRAMP-C1). The results of this research revealed that the effect of PP on the apoptosis of tumor cells was mediated through the raising of the Bax/Bcl2 expression ratio and the activation of caspase-3. The antiproliferative activity on human bladder cancer T24 cells was also associated with the ellagic acid [51]. In another study, Fazio et al. [38] proved that the β-glucan isolated from PP exhibited an antiproliferative activity against human breast MCF-7 and uterine HeLa cancer cells. Overall, the findings found in these studies suggest that PP extracts could be used as a promising drug, oriented towards the treatment and prevention of different types of cancer.

Punicalin, punicalagin and ellagic acid are also effective in inhibiting inflammatory processes. A recent study using an in vitro model of human colonic adenocarcinoma Caco-2 cells indicated that aqueous PP extract, at a dose of 10 µg/mL, resulted in a reduction in the secretion of CXCL8 (pro-inflammatory cytokine with chemotactic activity towards T lymphocytes, basophils and neutrophils) of 43%, compared to the positive control [76]. Stojanovic’ et al. [77] also suggested that the phytochemicals present in the skin of the pomegranate could have an important role in the treatment of autoimmune and chronic inflammatory illnesses, such as multiple sclerosis and type 1 diabetes. In another study, pectins isolated from PP regulated the immune system inflammatory response, through macrophages stimulation, natural killer cells, dendritic cells and T and B cells [35].

## 4. Role of Pomegranate By-Products in the Formulation of Meat Products

Meat is a complex system that contains water, proteins, lipids, minerals, and a small proportion of carbohydrates. Due to this rich nutritional profile, meat and meat products are easily susceptible to microbial growth and oxidation reactions (degradation of lipids, proteins and pigments), causing the loss of their quality attributes during processing and storage [4]. Moreover, microbial spoilage in meat contributes to the development of unattractive odors and flavors, discoloration, gas formation, and the visual perception of slime [4]. To overcome these problems, both the meat industry and academic researchers have proposed different alternatives for improving the quality and shelf life of meat and processed meat products [2].

Synthetic antioxidants have been commonly applied in the food industry to both reduce oxidative deterioration and inhibit the growth of spoilage bacteria in meat products [2,7]. However, the use of these synthetic preservatives should be limited due to their toxicity and carcinogenicity, as reported in several studies [78,79]. The substitution of synthetic additives with natural antioxidants is a suitable strategy for maintaining the sensory and microbiological quality, and extending the shelf life, of meat products [2,4,7]. In this context, the incorporation of pomegranate by-products, which contain high amounts of phenolics, flavonoids and tannins with excellent antioxidant properties, could replace artificial additives for preserving perishable foods. Besides, pomegranate by-products are a good source of dietary fiber, which, besides improving the physicochemical properties of meat products, also enrich their nutritional value, conferring functional properties that are health-promoting. 

One meat product, that has been reformulated with pomegranate by-products and studied by several researchers, is meatballs. For instance, Turgut et al. [80] evaluated the addition of pomegranate peel extract (PPE) to delay lipid and protein oxidation in this meat product. In their study, the pomegranate extracts were incorporated at 0.5% and 1%, and the results were comparable with those obtained using a synthetic antioxidant (BHT at 0.01%) and a control experiment (without any antioxidant) during 8 days of refrigerated storage. The authors found that beef meatballs elaborated with PPE exhibited greater lipid and protein stability, as well as enhanced sensory scores. In particular, the TBARS values recorded at the end of the storage were 1.19, 0.71, 0.60 and 0.56 mg MDA/kg for control, 0.5% PPE, BHT, and 1% PPE, respectively. Regarding the protein, oxidation was evaluated by the protein carbonyl and sulfhydryl levels. The reduction percent of protein carbonyl content was 25% for 1% PPE and BHT, and 12% for 0.5% PPE, as compared to control. In the case of sulfhydryl levels, the highest value (40.17 nmol/mg protein) was obtained for 1% PPE, and the minimum value was for the control sample (24.53 nmol/mg protein). Besides, the sensory evaluation indicated that the inclusion of PPE maintained the dark red or cherry red coloration, and prevented the formation of rancid odors in meatballs, throughout the 8 days of refrigerated storage. These authors also reported the same protective effect of PPE in beef meatballs during frozen storage for 6 months [81]. In general, the findings reported demonstrate that the high content of phenolic compounds in PPE, as well as their high antioxidant capacity, exert a protective effect against lipid and protein oxidation in meat products, even superior to that of BHT.

In an effort to extend the shelf life and enhance the safety of beef meatballs, Morsy et al. [82] applied lyophilized pomegranate peel nanoparticles (LPP-NPs) as a natural antioxidant and antimicrobial in their formulation. After 15 days of storage at 4 °C, the indicators of protein degradation (total volatile base nitrogen) and lipid peroxidation (peroxide value and TBARS) of the meatballs containing 1.5% LPP-NPs were lower than those recorded for samples with BHT (0.01%), and for the control without antioxidants. As well, LPP-NPs-treated meatballs presented less microbial growth during storage, attributed to the presence of phenolics and tannins in the LPP-NPs, which exert antimicrobial activity. The results also revealed that the incorporation of LPP-NPs improved the water holding capacity of beef meatballs. This positive effect could be associated with the presence of fiber in the pomegranate peels that acts as a water-binding agent. As in the works previously described, the LPP-NPs-based meatballs showed good acceptance, with high scores in terms of color and odor up to 15 days in refrigerated storage.

Recently, Fourati et al. [83] investigated the impact of pomegranate peel ethanol extract (PPEE) at three different dosages (0.1%, 0.5% and 1%) on the microbiological, oxidative stability and sensory attributes of minced beef meat. After 21 days of refrigerated storage, the thiobarbituric acid reactive substances (TBARS) values were significantly lower (*p* < 0.05) in meat samples with 1% PPEE than those found for the control without antioxidant. The results also indicated that this same extract dose led to a reduction in MetMb (56.68%), the formation of carbonyl groups (65.71%) and the loss of sulfhydryl groups (59.69%), in comparison with the control. Besides, PPEE inhibited the growth of spoilage microorganisms, exhibiting a dose-dependent protective effect. Finally, sensory evaluation, concerning the attributes of color, appearance and odor, as well as the global acceptability, revealed that the meat treated with 1% PPEE had the highest scores. In previous research, Qin et al. [84] also reported that pomegranate rind powder extract (PRPE), at a dose of 0.02 g extract/100 g meat, displayed a protective effect against lipid oxidation in the raw ground pork meat. Despite the fact that the treated samples presented changes in color and odor, the overall acceptability was higher than in the control group.

Other reformulated meat products based on pomegranate were frankfurters and cooked sausages. For example, Firuzi et al. [85] incorporated PRPE at 10 mg gallic acid equivalent/100 g in frankfurters, and they observed that the treated samples presented higher stability to lipid oxidation. By the end of the storage period (60 days at 4 °C), PRPE significantly (*p* < 0.05) reduced the peroxide value by 65.05% and 59.22%, respectively, in comparison with the control group (without additives). Use of PRPE, as well as BHT and nitrite, resulted in a raising of the lightness, and in a decreasing trend in the redness and yellowness values, in all frankfurter samples. Furthermore, samples containing antioxidants (natural or synthetic) resulted in less color variation (ΔE), indicating their effectiveness in delaying the discoloration of frankfurters during their refrigerated storage.

In another study, cooked sausages made with two concentrations (5‰ and 10‰) of a commercial mix of pomegranate and citrus extracts (Naturmix WM^®^, MEC Import, Rome, Italy) resulted in a significant decrease (*p* < 0.01) of TBARS values during refrigerated vacuum storage. Regarding sensory analysis, the findings denoted that the addition of natural extracts prolonged the shelf life of cooked sausages by up to 60 and 50 days, for doses of 10‰ and 5‰, respectively, compared to the control batch (42 days) [86].

The use of pomegranate by-products in beef burgers [87], and both lamb and beef patties [88,89], has also been evaluated by other authors. Shahamirian et al. [87] reported that the reformulation of beef burgers with pomegranate rind powder extract (PRPE) at a level of 0.01% resulted in remarkably lower TBARS values, retarded the aerobic bacterial count, and had a positive effect on color stabilization, as compared to the control sample during frozen storage for 90 days. Equally, burgers containing PRPE had the highest scores in terms of color, flavor, odor, texture and total acceptance. In another study, Bouarab-Chibane et al. [89] also observed that the inclusion of pomegranate peel at 10 g/kg in beef patties inhibited lipid and myoglobin oxidation, and delayed color variation, at the end of storage in a high oxygen atmosphere for 12 days at 4 °C. However, the authors observed that the burgers containing PP had a drier texture, which was attributed to the high amount of added extract. Contrary to the results of these studies, Andrés et al. [88] found that the addition of aqueous pomegranate by-product extracts (1000 mg/kg) in lamb patties led to TBARS and free thiol values similar to the control treatment. Based on the microbial analysis, the pomegranate extracts showed a strong inhibitory effect on the mesophilic and psychotropic counts, concluding that these extracts could be applied as natural antimicrobial additives in order to prolong the shelf life of lamb.

In an attempt to improve both the nutritional profile and the sensory and technological attributes of meat products, several studies have focused on the development of functional muscle foods based on antioxidant dietary fiber [1]. Moreover, the dietary fiber (including also pectin) has been reported for its ability to retain water, which might provide the lubricity and melting sensation to low-fat meat products [90]. For instance, Sharma et al. [91] elaborated chicken meat patties by adding pomegranate peel (2%) or pomegranate peel powder aqueous extract (6%). Both types of reformulated patties had a higher content of phenolics, fiber and ashes, providing additional nutritional benefits. Moreover, the water holding capacity, emulsion stability and cooking yield were slightly improved in the treated samples. As discussed in some of the previously cited works, the chicken patties with pomegranate peel exhibited a better protection against lipid oxidation and microbial deterioration during refrigerated storage, compared to samples treated with BHT. Similarly, Santhi et al. [92] proposed the fortifying effect of pomegranate pomace powder (PPP) in the elaboration of low-fat chicken meatballs. According to the authors, the inclusion of dietary fiber in chicken meat would be a suitable and low-cost choice for developing functional meat products with improved nutritional value.

Another study conducted by Devatkal et al. [93] evaluated the effect of vacuum packaging and PPE on the quality aspects of ground goat meat and cooked goat meat nuggets, over 25 days of refrigerated storage. Three treatments were studied: aerobic packaging (AP), vacuum packaging (VP) and vacuum packaging with PPE at 1% (VP + PPE). Samples treated with PPE showed greater stability to lipid oxidation, with significantly lower TBARS values than AP or VP. The addition of PPE reduced the TBARS level by 41% in ground meat and 40% in nuggets. The results found in this work indicated that the combination of PPE and VP is an adequate strategy for prolonging the shelf life of goat meat and nuggets.

Table 2 collects the research works developed in the last 10 years on the incorporation of pomegranate products in meat processed foods, and their main effects.

## 5. Conclusions and Future Trends

Pomegranate fruit consumption has increased in the last decades due to their excellent health benefits, and their generating of huge amounts of by-products that are usually discarded or underutilized. However, these residues contain remarkable amounts of bioactive compounds with potential functional properties. In particular, PP has been outlined as a rich, promising source of biomolecules with demonstrated bioactivities, such as antioxidant, antimicrobial, anti-inflammatory, antihyperglycemic, antihyperlipidemic and anticancer, making it suitable to be used as a natural additive in foodstuffs. In this context, the meat industry seeks alternatives to synthetic preservatives for guaranteeing stability and extending shelf life at the same time, which would result in more healthy meat processed products. The studies collected in this review revealed that the incorporation of PP and its extracts in these products is a suitable strategy for enhancing their quality attributes and functional properties. This approach opens a new field in the meat sector: to functionalize elaborated meat products tailored to the new consumers’ demands. Moreover, the reuse of this agro-food residue in another food industry will contribute to a circular economy model based on sustainable processes. However, more future research, focused on the bioavailability of the bioactive compounds from PP added to meat products, are imperative in order to prove their health effects.

## Figures and Tables

**Table 1:** Extraction technologies to obtain bioactive compounds from pomegranate peels, as well as the analytical techniques reported for their identification.

Technology	Extraction Conditions	Identification Method	Outcomes	Reference
SLE	Methanol: water (4:1, *v*/*v*) for 3 days at room temperature, LSR of 5:1 (mL/g)	HPLC-PDA	TPC: 188.9 mg GAE/g dw; TFC: 13.95 mg QE/g dw Identification of 23 phenolic compounds and 20 flavonoids	[43]
CPE	For total phenols: 8.22% Triton X-114, 4% NaCl at 36.80 °C and pH 4 For flavonoids: 8.27% Triton X-114, 4.06% NaCl at 34.30 °C and pH 5.07	Not indicated	TPC: 205.2 mg of GAE/g; TFC: 60.05 mg of QE/g	[59]
SLE, IR and UAE	Solvents: water, 50% ethanol and 8 different DES. The temperature for the three technologies was fixed at 50 °C	HPLC-DAD	The combination of DES and IR leads to the highest yield of polyphenols (152 mg/g) with high antioxidant activity and good antimicrobial properties. Identification of caffeic acid, kaempferol, luteolin, protocatechuic acid, ellagic acid, chlorogenic acid, hydroxybenzoic acid, gallic acid and quercetin	[60]
MAE and UAE	For MAE: 50% aqueous ethanol; LSR: 60:1 mL/g; power, 600 W for 4 min For UAE: water, LSR: 32.2:1 mL/g, amplitude level: 39.8%, pulse duration/pulse interval ratio, 1.2/1 at 34.7 °C for 10 min	HPLC-UV–vis	TPC (MAE): 199.4 mg GAE/g; TPC (UAE): 119.82 mg GAE/g. Identification and quantification of punicalagin: 143.64 mg/g for MAE and 138.8 mg/g for UAE	[25]
MASE	TPC: 600 W, 24% ethanol for 35 min; TFC: 800 W, 30% ethanol for 25 min; TTC: 800 W, 30% ethanol for 15 min	UHPLC/ESI/MS	TPC: 373 mg GAE/g; TFC: 155 mg RE/g; TTC: 317 mg GAE/g. Identification of 13 phenolic compounds, mainly ellagitannins and granatin	[61]
PUAE	Intensity level: 105 W/cm^2^; duty cycle: 50% for 10 min	HPLC-UV-DAD	Punicalagin: 146.58 mg/g; ellagic acid: 20.66 mg/g; gallic acid: 0.053 mg/g	[62]
UAE	Ultrasonic amplitude 60% for 6.2 min	Not indicated	Yield: 13.1%; TPC: 42.2 mg GAE/g; DPPH: 88%; FRAP: 1824.6 μmol Fe^2+^/g; IC50: 0.51 mg/mL	[23]
UAE	Ultrasonic power of 148 W, 55 °C for 63 min using LSR: 24 mL/g	Not indicated	Polysaccharide yield: 13.658%	[63]
HIFU	Choline chloride:acetic acid:water (1:1:10 M), amplitude of 60% for 11 min	HPLC-ESI-Q-TOF/MS	Protein: 20 mg/g. Identification of 23 different peptides and of 20 different phenolic compounds, mainly punicalin, punicalagin, galloyl-HHDPhexoside, ellagic acid-hexoside, and ellagic acid	[29]
HPE	Depending on the variable evaluated, the conditions ranged between 356 and 600 MPa and 32–80% ethanol for 23–30 min	UHPLC-DAD/LC-DAD/ESI-MS	Yield: 31 mg/g; TPC: 52 mg GAE/g; TFC: 20 mg of QE/g; TTC: 2.02 mg of CAE/g; TAC: 86 mg cyd-3-glu/g; ABTS: 269 mg TE/g; DPPH: 314 mg TE/g; FRAP: 436 mg TE/g	[64]
HPE + EE	HPE conditions: 300 MPa for 15 min; EE: 4% pectinase and 4% cellulase for 15 min	HPLC-DAD-MSn	Total extraction yield: 41%; TPC: 207 mg GAE/g; DPPH: 334 mg TE/g Identification of quinic acid, a punicalin isomer, two bis-HHDP-glucoside isomers, 2-O-galloylpunicalagin, two punicalagin isomers, galloyl-HHDP-glucoside and digalloylpentoside	[24]
EASCFE	EE: cocktail enzyme (mixture of cellulase, pectinase and protease; 50:25:25) at 3.8%, 49 °C, pH 6.7 for 85 min; SCFE: ethanol as co-solvent (2 g/min), 55 °C, 300 bar for 100–120 min	HPLC-DAD-ESI–MS	TPC: 301.53 mg GAE/g Identification of p-coumaric acid, vanillic acid, gallic acid, caffeic acid, ferulic acid, syringic acid, sinapic acid	[30]

TPC: total phenolic compounds; TFC: total flavonoid compounds; TTC: total tannin content; TAC: total anthocyanins content; GAE: gallic acid equivalents; QE: quercetin equivalents; RE: rutin equivalents; CAE: catechin equivalents; TE: Trolox equivalents; cyd-3-glu: cyanidin-3-glucoside equivalents; CPE: cloud point extraction; SLE: solid–liquid extraction; DES: deep eutectic solvents; MAE: microwave-assisted extraction; MASE: microwave-assisted soxhlet extraction; PUAE: pulsed ultrasound-assisted extraction; UAPLE: ultrasound and pressurized liquid extraction; HIFU: high intensity focused ultrasounds; HPE: high pressure extraction; EE: enzymatic extraction; EASCFE: enzyme-assisted supercritical fluid extraction.

**Table 2:** Meat products reformulated with pomegranate peel extracts.

Meat Product	Material	Amount Used	Storage Conditions	Main Effects	References
Beef meatballs	PPAE	0.5% and 1%	Refrigerated at 4 °C for 8 days	Decrease of lipid and protein oxidation. Prevents rancid odor formation. Improved shelf life	[80]
			Frozen at −18 °C for 6 months		[81]
	LPP-NPs	1% and 1.5%	Refrigerated at 4 °C for 15 days	Decreased peroxide, TBARS and total volatile base nitrogen contents. Improved microbiological quality. No negative impact on sensory properties	[82]
Minced beef meat	PPEE	0.1%, 0.5% and 1%	Refrigerated at 4 °C for 21 days	Reduction of oxidative deterioration. Inhibition of growth of spoilage microorganisms. Higher score for color, appearance, odor and overall acceptability	[83]
Raw ground pork meat	PRPE	0.02%	Refrigerated at 4 °C for 12 days	Reduction in peroxide and TBARS values. Decreased lightness value. Better overall acceptability of the treated samples	[84]
Frankfurter	PRPE	10 mg GAE/100 g	Refrigerated 4 °C for 60 days	Reduction in peroxide and TBARS values. Increase in L* value and reduction in a* and b* values	[85]
Cooked sausages	Naturmix WM^®^	5‰ and 10 ‰	Refrigerated at 4 °C for 60 days in vacuum-packaged	Delay of the growth of total viable count, psychrotrophic microbial counts and *Lactobacillus* spp. Better acceptability of the product. Enhanced shelf life	[86]
Beef burgers	PRPE	0.01%	Frozen at −18 °C for 90 days	Retarded lipid oxidation. Reduced growth of aerobic bacteria. Improved the overall acceptability of the product	[87]
Beef patties	PP	10 g/kg	Refrigerated at 4 °C for 12 days in a high O_2_ atmosphere	Reduction in TBARS levels. Decrease redness value. Modified the texture and taste	[89]
Lamb patties	POM	1000 mg/kg	Refrigerated at 2 °C for 7 days	Reduced the growth of mesophile bacteria and psychrotrophic bacteria	[88]
Chicken patties	PP/PPAE	2% and 6%	Refrigerated at 4 °C for 16 days	Increased the content of phenolics, fiber and ashes. Improved the water holding capacity, emulsion stability and cooking yield. Retarded lipid oxidation and microbial deterioration.	[91]
Low-fat chicken meatballs	PPP	0.5%, 1%, 1.5% and 2%	Not indicated	Improved the fiber level. Decrease of sensory scores with the increase % of PPP	[92]
Ground goat meat and cooked nuggets	PPE	1%	AP and VP for 25 days at 4 °C	Reduction in TBARS content. Extended shelf life	[93]
Meat paté	PPE	7.5% (*v*/*v*)	Refrigerated at 4 °C for 46 days	Inhibition of *Listeria monocytogenes*	[94]

PP: Pomegranate peel; PPAE: Pomegranate peel aqueous extract; LPP-NPs: Lyophilized pomegranate peel nanoparticles; PPEE: Pomegranate peel ethanol extract; PRPE: Pomegranate rind powder extract; GAE: gallic acid equivalent; Naturmix WM^®^: Commercial mix of pomegranate and citrus extracts; POM: Pomegranate extract; PPP: pomegranate pomace powder. AP: aerobic packaging; VP: vacuum packaging.
